# Dimensions and predictors of disability—A baseline study of patients entering somatic rehabilitation in secondary care

**DOI:** 10.1371/journal.pone.0193761

**Published:** 2018-03-02

**Authors:** Vegard Pihl Moen, Jorunn Drageset, Geir Egil Eide, Sturla Gjesdal

**Affiliations:** 1 Center for Habilitation and Rehabilitation, Haukeland University Hospital, Bergen, Norway; 2 Department of Global Public Health and Primary Care, University of Bergen, Bergen, Norway; 3 Center for Clinical Research, Haukeland University Hospital, Bergen, Norway; IRCCS E. Medea, ITALY

## Abstract

**Purpose:**

The purpose of this study was to investigate disability among patients who were accepted for admission to a Norwegian rehabilitation center and to identify predictors of disability.

**Materials and methods:**

In a cross-sectional study including 967 adult participants, the World Health Organization Disability Assessment Schedule version 2.0 36-item version was used for assessing overall and domain-specific disability as outcome variables. Patients completed the Hospital Anxiety and Depression Scale (HADS), EuroQoL EQ-5D-5L and questions about multi-morbidity, smoking and perceived physical fitness. Additionally, the main health condition, sociodemographic and environmental variables obtained from referrals and public registers were used as predictor variables. Descriptive statistics and linear regression analyses were performed.

**Results:**

The mean (standard error) overall disability score was 30.0 (0.5), domain scores ranged from 11.9 to 44.7. Neurological diseases, multi-morbidity, low education, impaired physical fitness, pain, and higher HADS depressive score increased the overall disability score. A low HADS depressive score predicted a lower disability score in all domains.

**Conclusions:**

A moderate overall disability score was found among patients accepted for admission to a rehabilitation center but “life activities” and “participation in society” had the highest domain scores. This should be taken into account when rehabilitation strategies are developed.

## Introduction

Disability is a complex phenomenon affecting many aspects of an individual’s life, including common daily activities and participation in society, and it affects the individual’s quality of life. Prevalence figures for disability vary. In 2011, the World Health Organization (WHO) estimated that 15% of the world’s population lives with some form of disability and that this prevalence is increasing [1]. A precise definition of the concept of disability is lacking. The model of disability in the International Classification of Functioning, Disability and Health (ICF) [2] emphasizes the complexity, showing multifactorial determinants, including the interaction between health conditions and contextual factors with effects on impairment, activities, and participation in society.

The prevalence of disability increases with age [3–5]. Women report more functional limitations and a higher degree of disability compared with men [6–11]. Additionally, an association between disability and marital status has been reported [12]. Higher educational level leads to better outcomes [7, 12], and living in rural areas is associated with higher disability compared with living in urban areas [13]. Poorer health and higher distress cause higher disability (i.e., multi-morbidity, impaired physical health, pain, and depressive symptoms) [7, 13–15]. For symptoms of anxiety, the association with disability is not conclusive [7, 16].

Although the ICF was released in 2001, many of the above-mentioned studies conceptualize disability according to the medical model. Instruments that were developed from the 1970s and later, such as the Katz Index of Activities of Daily Living (1970) [17] and the Hospital Assessment Questionnaire (1980) [18], are still in use. Primary daily activities are often assessed in the concept of disability, while items concerning participation in society are seldom included in surveys [19]. The choice of model constituting the basis of a study is essential when investigating predictor variables because variables vary with disability domains [11].

The WHO Disability Assessment Schedule 2.0 (WHODAS 2.0) is based on the ICF model and was developed through a comprehensive process [20]. This instrument consists of an overall score and scores on the following dimensions: Cognition, Mobility, Self-care, Getting along, Life activities, and Participation in society. The WHODAS 2.0 has been translated into many languages, including Norwegian, and has been validated in various settings and countries, including specialized somatic rehabilitation [21].

Few studies have been conducted to assess determinants of disability conceptualized in the ICF among patients who are accepted for rehabilitation, even in secondary care. One previous study including people who applied to a disability registration system, investigated sociodemographic/socioeconomic variables and the type and severity of impairment as predictors for disability [22]. The investigated group was eligible for disability benefits, but it is not clear whether the individuals in the study sample were accepted for rehabilitation, and people with musculoskeletal disorders, which is a large group in rehabilitation settings, were not included. Knowledge of determinants that are associated with disability is important for identifying subgroups for implementing preventive and treatment strategies [1, 3, 23], including rehabilitation settings.

In Norway, the Parliament has developed a national strategy for rehabilitation with the aim of providing disabled people with the tools to regain optimal functioning, health, and well-being. Primary care provides rehabilitation in municipalities to patients where long-term follow-up and competence related to the local community are required, with focus on the elderly population. Secondary care provides rehabilitation to patients with complex health issues in hospitals and rehabilitation centers. The characteristics of this service are comprehensive inter-professional interventions with a high degree of competence, methodology and infrastructure. In hospitals, rehabilitation is secondary to medical treatment which is the primary goal of admittance. Patients admitted to the rehabilitation centers should be stable after medical treatment. Access to the Norwegian rehabilitation centers occurs after assessment of a general practitioner’s referral or after elective or emergency hospitalization is completed.

The present study aimed to provide new knowledge on this patient group, to improve rehabilitation services. The aim was to present the overall disability scores and domain scores among these patients. Furthermore, the study also investigated associations between the overall disability score as measured by the WHODAS 2.0 and its dimensions, and sociodemographic factors, multi-morbidity, medical condition (diagnosis), physical fitness, pain, and symptoms of depression and anxiety.

## Materials and methods

### Design, sample, and procedure

The study used data from a cross-sectional study of patients living in the Western Norway Health Region who were accepted for admission to a rehabilitation center. Data were collected between January 2015 and July 2015 as a baseline for a prospective cohort study surveying patients before admittance and after discharge from a rehabilitation center. All referrals from primary care are treated by a regional assessment team. Referrals from hospitals are sent directly to the rehabilitation center.

The patients were invited by mail from a waiting list or at admittance in the following rehabilitation centers: Åstveit Health Center, Red Cross Haugland Rehabilitation Center, Ravneberghaugen Rehabilitation Center, LHL Clinics Bergen, LHL Clinics Nærland, and Rehabilitering Vest Rehabilitation Center. Patients were included if they were at least 18 years old and had sufficient knowledge of the Norwegian language to complete a questionnaire. Patients who were referred for a follow-up stay and those who were referred to rehabilitation because of morbid obesity were excluded.

Patient-reported data were collected. For invitations by mail, a reminder was sent after 1 month. For patients who were invited to participate in the study at a rehabilitation center, the questionnaires were completed within the first 2 days after admittance, with no reminders. The main health condition (ICD-10 chapters) leading to referral was collected from the medical records.

Individual data on educational attainment, municipality of residence, and civil status, which were retrieved from public registers, were linked to survey data by Statistics Norway based on each patient’s written consent.

### Ethics

The study was approved by the Regional Ethics Committee West in Norway (REK-No. 2014–1636). Informed and written consent was obtained from all participants in the study.

### Instruments

The survey package consisted of the WHODAS 2.0 [20], the Hospital Anxiety and Depression Scale (HADS) [24], and the EuroQol EQ-5D-5L [25]. The patients were also asked about smoking, physical fitness, physical activity, coinciding chronic conditions, and health care use.

### Outcome variables

The WHODAS 2.0 36-item version is a generic, patient-reported instrument that measures health and disability based on the ICF [26]. The Norwegian version of this instrument has been tested for its psychometric properties in rehabilitation services, with satisfactory reliability and moderate validity [21]. This instrument assesses disability during the last 28 days (30 in the original) in six functional domains. These domains are Cognition (6 items), Mobility (5 items), Self-care (4 items), Getting along (5 items), Life activities (8 items), and Participation (8 items). Life activities consist of activities related to the household (4 items) and activities related to work or study (4 items). The patient scores each item on a 5-point Likert scale with two anchor responses of “none” and “extreme or cannot do”. Scores for each domain and an overall disability score were calculated according to the manual using “complex scoring” [26], with range from 0 (no disability) to 100 (full disability). For people working or studying, all 36 items were calculated for an overall score. Otherwise, four items were omitted and 32 items were computed as an overall score. An algorithm enabled calculation of the domain score of Life activities and the total score, regardless of whether the four items related to work or study were answered. In this study, all of the domain scores and the overall score were used as outcome variables.

### Predictor variables

Age was categorized by decades.

Health conditions were divided into musculoskeletal, circulatory, and neurological diseases, neoplasms, endocrine, nutritional, and metabolic diseases, respiratory diseases, injuries and external causes, factors influencing health status and contact with health services, mental and behavioral disorders and miscellaneous. Miscellaneous conditions were as follows: symptoms, signs, and abnormal clinical and laboratory findings, not elsewhere classified (n = 9); codes for special purposes (n = 7); diseases of the digestive system (n = 6); diseases of the blood and blood-forming organs, and certain disorders involving the immune mechanism (n = 5); diseases of the ear and the mastoid process (n = 3); diseases of the genitourinary system (n = 3); congenital malformations, deformations, and chromosomal abnormalities (n = 3); and certain infectious and parasitic diseases (n = 2). In regression analyses, health conditions with n < 50 were merged with the miscellaneous conditions into one category, “other”.

Multi-morbidity was defined as two or more coinciding chronic diseases or conditions by the same individual [27]. In addition to the referral diagnoses, one or more of the following diseases were reported: heart attack, angina pectoris, heart failure, other heart disease, stroke/cerebral hemorrhage, kidney disease, asthma, chronic bronchitis/emphysema/chronic obstructive pulmonary disease, diabetes, cancer, epilepsy, rheumatoid arthritis, Bechterew’s disease, sarcoidosis, osteoporosis, fibromyalgia, arthrosis, and psychological problems (which have been consulted for previously). Any case of missing data was defined as an absence of the disease in question.

Admission was dichotomized as initial (referred from primary care) or ongoing management (referred from hospital).

Marital status was dichotomized to married and not married. Educational attainment was categorized as primary school, high school, and college/university. Smoking status was dichotomized to current smoking or non-smoking. Living area was dichotomized to rural and urban with a cutoff of 20,000 inhabitants in the municipality.

Physical fitness was measured by a single question with two anchor responses of “very poor” and “very good”. Three categories were chosen: poor (merging very poor and poor), moderate, and good (merging very good and good).

Pain/discomfort was assessed using the EQ-5D (-5L) [25]. This instrument consists of five questions and a health rating scale. The questions assess physical activities, psychological distress, and pain/discomfort. For pain/discomfort, the score ranges from no pain/discomfort to extreme pain/discomfort, with a total of five responses. This instrument has been tested extensively for its measurement properties, among others in chronic conditions [28].

Depression and anxiety scores were assessed using the HADS [24]. This instrument forms two subscales, depression (HADS-D) and anxiety (HADS-A), with seven questions each with responses being scored on a scale of 0–3. For each subscale, the score ranges from 0–21 (higher score for higher severity). Scores for patients with less than three missing questions per subscale were included, and scores were imputed based on the mean across each person’s available responses in each subscale. The HADS performs well as a screening instrument in assessing the severity of symptoms in somatic patients [29], shows adequate measurement properties in terms of validity and reliability, and a two factor-structure model is supported [30].

### Statistical analysis

The mean/median and standard error (SE) of the WHODAS 2.0 overall score and scores of the six domains were estimated according to categories of the different predictors. For two-group comparisons we used the exact chi-square test for categorical variables and the Mann-Whitney test for continuous variables. Analysis of variance (ANOVA) with F-test was performed to investigate differences in disability scores in variables with more than two categories. Tukey’s post hoc test was used for subgroup comparisons. The relative risk of pain/discomfort related to sex was calculated.

The overall disability score and score of the domains were analyzed separately as response variables in linear regression models with the following predictor variables: sex, age, health condition, multi-morbidity, marital status, education, smoking, living area, physical fitness, HADS-D score, and HADS-A score. The EQ-5D (pain/discomfort), HADS-D, and HADS-A scores were treated as continuous variables, and the other variables as categorical variables.

Linear regression was first performed with one predictor variable at a time, and then with all predictor variables included simultaneously. Interactions were tested between health conditions, multimorbidity and physical fitness. For domains, only adjusted results are presented. Results are reported as the estimated regression coefficient (b), the SE or 95% confidence interval (CI), and p value from the F-test.

Missing items were treated according to the WHODAS 2.0 manual by using multiple imputations [26]. WHODAS 2.0 data were excluded if the rate of missing items was > 50% in domains or in the total score. The number of imputation sets was five. The significance level was chosen as 0.05 throughout. IBM SPSS for Windows version 23.0 (IBM Corp., Armonk, NY) was used for all statistical analyses.

## Results

**Fig 1:**
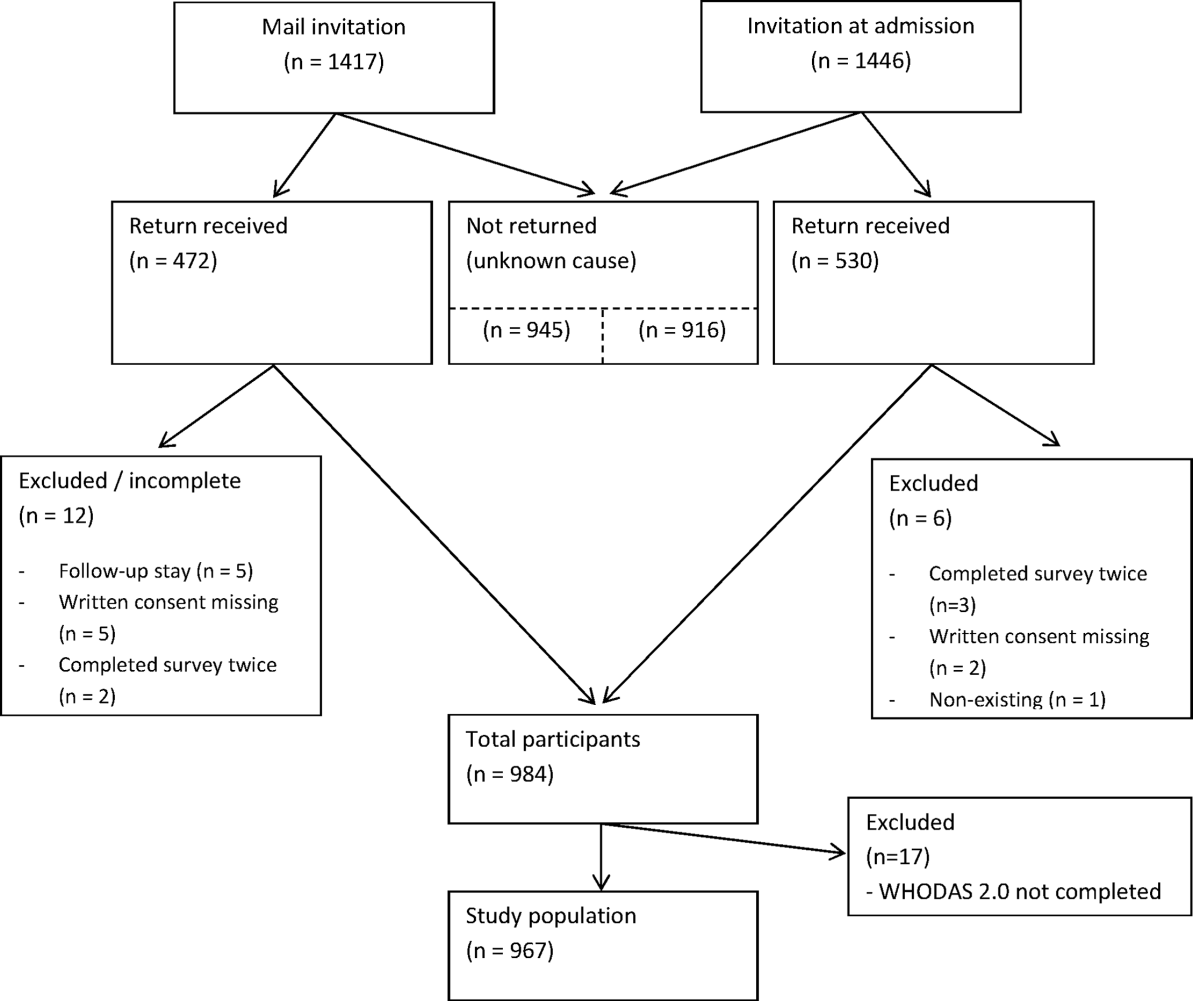
Patients accepted for rehabilitation in the Western Norway Health Region in January 2015 –July 2015.

A total of 3226 patients, living in the Western Norway Health Region, were accepted for admission to a rehabilitation center between January and July 2015, and 2863 were invited (1885 women and 978 men). Of these, 984 returned the questionnaire with signed consent and fulfilled the inclusion criteria, and 967 completed at least 50% of the items in the WHODAS 2.0. Therefore, the overall response rate was 34.6%, with 32.6% for women and 36.6% for men. Response rates for patients who were recruited per mail and at admission to rehabilitation centers were 32.7% and 36.8%, respectively. The lowest response rate was among those aged 18–29 years (17.7%) and > 80 years (20.7%), and the highest response rate was for patients aged 60–69 years (44.1%). Fig 1 shows details of the recruitment procedures.

### Characteristics of participants

The mean age (standard deviation: SD) of participants was 57.6 (14.0) years and 63.2% were women.

The mean/median HADS-D and HADS-A scores were 5.3/5.0 and 6.0/5.0, respectively, on a scale ranging from 0–21 (maximum distress).

Among the participants, 7.4% reported no pain/discomfort, 30.2% reported slight pain/discomfort, 33.4% reported moderate pain/discomfort, 24.2% reported severe pain/discomfort, and 4.7% reported extreme pain/discomfort. The female to male ratio was 1.37 for extreme pain/disability, 1.47 for severe pain/discomfort, 1.45 for moderate pain/discomfort, 0.63 for slight pain/discomfort and 0.39 for no pain/discomfort.

Women had a higher prevalence of multi-morbidity, a higher proportion of women were current smokers, fewer women were married, and women had a higher HADS-A-score compared with men (all p < 0.05).

A significantly higher proportion of non-participants (67.2%) was women compared with participants (63.2%), (p < 0.05). The mean age (SD) of non-participants, 55.6 (16.7) years, was significantly lower than that of participants, 57.6 (14.0) years, (p < 0.001).

There were larger proportions of women, musculoskeletal diseases, married, and smokers among patients with initial rehabilitation (all p-s<0.05), while there were more patients with circulatory diseases in the group of ongoing management (p<0.05).

### Missing data

The percentages of missing values were 0.9% for education, 5.8% admission, 0.3% for marital status, 1.9% for physical fitness, 4.1% for EQ-5D pain/discomfort, 1.2% for the HADS-D score, and 1.4% for the HADS-A score. The proportion of missing items for the WHODAS 2.0 ranged between 0.6% and 3.1% for the various domains, with the highest proportion of missing items in Participation and the lowest for Self-care. The item concerning sexual activity was missing for 10.3% of participants. The proportion of missing data for the other items ranged between 0.3% and 5.5%.

### Disability scores

Table 1 shows the overall and domain disability scores according to the predictor variables. The mean (SE) overall disability score was 30.0 (0.5) and differed between the age groups (ANOVA p < 0.001). Patients aged from 40–49 years had the highest overall disability score, which was significantly higher than that of patients aged 50–59 and 60–69 years (p = 0.002 and p = 0.001 respectively). The overall disability score differed also between the health conditions (ANOVA p < 0.001). Patients with neurological diseases reported the highest overall disability, which was significantly higher than that for respiratory diseases, factors influencing health status and contact with health services and circulatory diseases (p = 0.002, p < 0.001 and p < 0.001, respectively). Also, there were differences between the educational level groups (ANOVA p < 0.05). Patients with primary school education scored significantly higher on overall disability compared with those with secondary school and college/university education (p = 0.0034 and p = 0.002, respectively). However, there was no significant difference in overall disability between patients with secondary school and those with college/university education. Also for physical fitness there were significant differences (p < 0.001). Patients who reported good physical fitness had a significantly lower overall disability score compared with patients who reported poor or moderate physical fitness (p < 0.001 for both). And patients who reported moderate fitness had significantly lower overall disability score compared with patients who reported poor physical fitness (p < 0.001).

**Table 1:** Distribution of overall and domain WHODAS 2.0 scores for disability^a^^)^ for patients accepted for rehabilitation.

Disability		Overall score			Domain scores					
Variables				Female	Cognition	Mobility	Self-care	Getting along	Life activities	Participation
*Categories*		n	Mean/median (SE)	(%)	Mean/median (SE)					
All		967	30.0/28.9 (0.5)	63.2	17.8/10.0 (0.6)	33.6/31.3 (0.8)	11.9/ 0.0 (0.6)	24.7/16.7 (0.7)	44.7/40.0 (0.9)	40.9/41.7 (0.7)
	*Men*	356	27.3/25.2 (0.8)	0.0	15.8/10.0 (0.9)	30.3/25.0 (1.4)	11.6/ 0.0 (0.9)	24.4/25.0 (1.1)	35.9/40.0 (1.5)	37.8/37.5 (1.1)
	*Women*	611	31.6/30.4 (0.6)	100.0	19.0/15.0 (0.8)	35.6/31.3 (1.0)	12.1/ 0.0 (0.7)	24.9/16.7 (0.9)	49.8/50.0 (1.1)	42.8/41.7 (0.8)
Age^b^^)^, *years*										
	*18–29*	27	32.9/31.5 (3.3)	76.9	28.1/20.0 (5.2)	33.5/25.0 (5.5)	13.8/10.0 (4.3)	29.2/25.0 (4.8)	43.8/40.0 (4.9)	43.6/45.8 (3.9)
	*30–39*	79	32.7/30.5 (1.7)	86.1	21.3/20.0 (2.0)	30.6/31.3 (2.7)	12.5/ 0.0 (2.1)	25.9/25.0 (2.5)	51.9/50.0 (3.1)	47.8/45.8 (2.3)
	*40–49*	180	34.1/34.6 (1.2)	69.3	25.0/20.0 (1.5)	32.6/31.3 (1.8)	11.9/ 0.0 (1.3)	32.4/33.3 (1.9)	49.1/50.0 (1.9)	47.5/47.9 (1.5)
	*50–59*	246	28.5/28.3 (0.9)	58.8	17.1/10.0 (1.2)	29.7/25.0 (1.6)	9.5/ 0.0 (1.0)	23.5/16.7 (1.3)	43.4/40.0 (1.7)	41.0/41.7 (1.2)
	*60–69*	235	27.2/26.7 (0.9)	57.8	13.9/10.0 (1.0)	31.7/25.0 (1.8)	10.9/ 0.0 (1.2)	22.5/16.7 (1.2)	40.4/40.0 (1.8)	36.6/33.3 (1.4)
	*70–79*	154	29.6/28.3 (1.2)	57.4	13.4/ 5.0 (1.5)	41.8/43.8 (2.2)	15.6/10.0 (1.6)	21.3/16.7 (1.4)	44.9/40.0 (2.5)	35.9/33.3 (1.9)
	*≥ 80*	46	30.6/28.0 (2.2)	59.1	13.3/ 9.0 (2.7)	46.9/43.8 (4.0)	15.8/10.0 (3.0)	20.0/16.7 (2.5)	41.9/40.0 (4.6)	38.5/33.3 (3.2)
Health condition, ICD-10										
	*Musculoskeletal diseases*	454	33.2/32.3 (0.7)	75.3	19.2/15.0 (0.9)	40.1/37.5 (1.1)	13.7/10.0 (0.9)	26.0/16.7 (1.0)	50.8/50.0 (1.2)	44.9/41.7 (1.0)
	*Circulatory diseases*	185	22.9/20.0 (1.1)	33.3	13.8/10.0 (1.2)	19.7/12.5 (1.7)	9.5/ 0.0 (1.2)	20.5/16.7 (1.3)	31.4/30.0 (2.0)	32.3/29.2 (1.5)
	*Neurological diseases*	83	34.2/33.8 (1.6)	54.9	17.9/10.0 (2.1)	44.1/43.8 (2.9)	16.2/10.0 (2.1)	27.7/25.0 (2.4)	54.3/50.0 (3.0)	45.1/45.8 (2.2)
	*Neoplasms*	50	32.2/29.3 (2.1)	83.7	25.7/20.0 (3.2)	27.2/25.0 (3.3)	8.4/ 0.0 (1.8)	31.7/33.3 (3.4)	49.4/40.0 (3.5)	43.1/41.7 (2.9)
	*Endocrine*, *nutritional*, *and metabolic diseases*	36	26.4/26.9 (2.4)	80.6	19.5/15.0 (3.4)	25.0/25.0 (3.8)	10.3/ 0.0 (3.0)	21.6/20.8 (3.4)	37.1/40.0 (4.0)	35.9/41.7 (3.0)
	*Respiratory diseases*	36	22.6/22.7 (2.1)	52.8	10.7/ 5.0 (2.2)	25.6/21.9 (3.9)	3.4/ 0.0 (1.6)	19.9/16.7 (3.0)	33.2/30.0 (4.6)	33.2/33.3 (3.3)
	*Injuries and external causes*	26	33.5/32.8 (3.7)	69.2	14.8/ 5.5 (4.1)	48.6/53.1 (6.6)	19.2/10.0 (4.8)	21.2/16.7 (4.0)	52.0/60.0 (7.4)	42.8/39.6 (5.0)
	*Skin diseases*	24	26.9/24.2 (3.0)	70.8	14.2/ 7.5 (3.6)	33.6/31.3 (5.0)	10.4/0.0 (4.0)	22.8/20.0 (3.8)	35.9/40.0 (5.1)	38.0/33.3 (3.9)
	*Factors influencing health status and contact with health services*	23	19.4/16.9 (3.0)	30.4	13.7/10.0 (2.8)	9.4/ 6.3 (3.8)	4.8/ 0.0 (3.5)	23.8/16.7 (4.9)	26.4/25.0 (5.3)	27.5/25.0 (3.7)
	*Mental and behavioural disorders*	12	29.1/27.2 (2.9)	66.7	25.5/25.0 (4.7)	19.9/ 6.2 (7.4)	5.8/ 0.0 (2.9)	27.1/25.0 (5.3)	36.7/35.0 (6.3)	46.7/44.2 (3.6)
	*Miscellaneous*	38	30.1/30.3 (2.1)	55.3	19.0/15.0 (3.1)	33.0/31.3 (4.3)	9.6/ 0.0 (2.1)	26.8/25.0 (3.6)	41.4/40.0 (3.6)	42.0/41.7 (3.3)
Multi-morbidity										
	*Yes*	619	32.0/31.1 (0.6)	65.6	19.7/15.0 (0.8)	35.5/31.3 (1.1)	13.8/10.0 (0.8)	26.7/25.0 (0.9)	47.5/50.0 (1.1)	43.1/41.7 (0.9)
	*No*	3489	26.5/25.0 (0.7)	58.5	14.3/10.0 (0.9)	30.1/31.3 (1.3)	8.5/ 0.0 (0.8)	21.1/16.7 (1.1)	39.7/40.0 (1.4)	37.1/37.5 (1.1)
Admission										
	*Initial*	644	32.0/31.1 (0.6)	65.6	19.2/15.0 (0.8)	33.3/31.3 (1.0)	11.0/ 0.0 (0.7)	26.5/25.0 (0.9)	45.7/50.0 (1.1)	42.6/41.7 (0.8)
	*Ongoing management*	267	26.5/25.0 (0.7)	58.5	13.8/10.0 (1.0)	32.5/25.0 (1.8)	13.2/ 0.0 (1.2)	20.0/16.7 (1.1)	41.2/40.0 (1.8)	35.8/33.3 (1.3)
Marital status										
	*Not married*	458	31.4/30.4 (0.7)	66.8	19.2/15.0 (0.9)	34.8/31.3 (1.3)	12.9/ 0.0 (0.9)	26.1/16.7 (1.1)	46.7/50.0 (1.3)	42.3/41.7 (1.0)
	*Married*	515	28.9/28.3 (0.7)	58.0	16.6/10.0 (0.8)	32.5/31.3 (1.1)	11.1/ 0.0 (0.7)	23.6/16.7 (0.9)	43.0/40.0 (1.2)	39.9/37.5 (0.9)
	*Unknown*	3	28.2/19.8 (8.3)	66.7	18.3/5.0 (14.4)	33.3/15.6 (16.7)	3.3/ 0.0 (3.3)	19.4/16.7 (8.2)	43.3/35.0 (10.9)	39.6/39.6(5.9)
Educational level										
	*Primary school*	198	32.9/32.2 (1.1)	68.8	21.4/15.0 (1.5)	39.5/38.8 (1.9)	14.5/10.0 (1.5)	25.4/16.7 (1.5)	46.6/40.0 (2.1)	44.1/41.7 (1.6)
	*Secondary school*	479	29.7/29.3 (0.7)	59.7	17.5/10.0 (0.9)	33.3/31.3 (1.2)	11.7/ 0.0 (0.8)	24.5/16.7 (1.0)	44.0/40.0 (1.3)	40.5/41.7 (1.0)
	*College/university*	277	28.2/26.1 (0.9)	66.1	15.4/10.0 (1.1)	29.7/25.0 (1.5)	10.2/ 0.0 (1.0)	24.9/25.0 (1.2)	44.3/40.0 (1.6)	39.1/37.5 (1.2)
	*Unknown*	9	34.4/35.9 (4.7)	38.5	23.0/10.0 (7.9)	37.0/31.3 (8.1)	16.9/ 0.0 (7.2)	23.1/16.7 (7.0)	47.7/40.0 (6.2)	48.1/45.8 (4.2)
Smoking										
	*Yes*	185	33.2/33.0 (1.0)	70.5	20.8/15.0 (1.4)	37.7/37.5 (1.9)	13.0/10.0 (1.2)	28.4/25.0 (1.6)	49.0/50.0 (2.0)	45.6/45.8 (1.4)
	*No*	773	29.3/27.4 (0.5)	65.7	17.1/10.0 (0.7)	32.5/31.3 (0.9)	11.6/ 0.0 (0.6)	23.8/16.7 (0.7)	43.7/40.0 (1.0)	39.8/37.5 (0.8)
Living area										
	*Urban*	508	30.5/29.3 (0.7)	65.0	17.8/10.0 (0.9)	34.6/31.3 (1.2)	12.6/ 0.0 (0.8)	24.8/16.7 (0.9)	46.5/50.0 (1.3)	41.4/41.7 (0.9)
	*Rural*	459	29.4/27.9 (0.7)	61.0	17.8/10.0 (0.9)	32.5/31.3 (1.2)	11.1/ 0.0 (0.8)	24.8/16.7 (1.0)	42.6/40.0 (1.3)	40.4/37.5 (1.0)
Physical fitness										
	*Poor*	413	35.3/34.5 (0.7)	64.9	21.4/15.0 (1.5)	42.6/43.8 (1.2)	15.6/10.0 (1.0)	29.1/25.0 (1.1)	53.0/50.0 (1.3)	47.2/45.8 (1.0)
	*Moderate*	375	27.9/26.1 (0.7)	63.7	17.5/10.0 (0.9)	29.1/25.0 (1.3)	9.5/ 7.4 (0.8)	22.8/16.7 (1.0)	41.1/40.0 (1.3)	39.0/37.5 (1.0)
	*Good*	163	21.3/18.7 (1.1)	56.1	15.4/10.0 (1.1)	20.7/12.5 (1.9)	8.1/ 0.0 (1.1)	18.0/13.3 (1.5)	31.0/30.0 (2.2)	29.9/29.2 (1.5)
	*Unknown*	18	28.5/20.3 (3.7)	72.2	13.8/10.0 (3.1)	31.9/21.9 (6.7)	8.3/ 0.0 (4.7)	25.5/25.0 (4.3)	48.8/50.0 (6.6)	36.3/33.3 (7.0)

*Abbreviations*: WHODAS: World Health Organization Disability Score; ICD-1: International Classification of Diseases version 10; SE: standard error of the mean.

^a)^ All scores: 0 = lowest score of disability, 100 = highest score of disability.

^b)^ Mean (standard deviation): 57.6 (14.0).

Mean scores for domains ranged between 11.9 and 44.7.

### Predictors for overall disability

Results from linear regression analysis for predicting the WHODAS 2.0 overall disability score are shown in Table 2. Except for living area, all predictor variables were significantly associated with disability in the unadjusted model (p < 0.05, i.e. men, initial rehabilitation, no smoking, being married, higher educational level, and better health significantly decreased the overall score). In the fully adjusted model, multi-morbidity, type of admission, education, physical fitness, the pain/discomfort item-score in EQ-5D, and the HADS-D score remained significant (all p < 0.05). Additionally, being referred with a neurological disease significantly increased the disability score compared with the other health conditions. No significant interactions were found, and the reported results are based on analyses with no interaction terms included in the statistical models.

**Table 2:** Linear regression analysis for predicting WHODAS 2.0 overall scores for patients accepted for rehabilitation.

Predictor variable		Unadjusted models			Adjusted model		
*Categories*		b	95% CI	p value	b	95% CI	p value
Intercept					1.37	(-2,32, 5.06)	
Female (ref: male)		4.32	(2.38, 6.27)	< 0.001	1.56	(-0.16, 3.29)	0.074
Age, years				< 0.001			0.134
	*18–29*	5.70	(-0.25, 11.64)		2.33	(-2.56, 7.13)	
	*30–39*	5.46	(1.71, 9.21)		0.03	(-3.13, 3.19)	
	*40–49*	6.91	(4.04, 9.77)		1.98	(-0.40, 4.36)	
	*50–59*	1.35	(-1.29, 3.98)		-0.85	(-2.98, 1.28)	
	*60–69*	0.00	(Reference)		0.00	(Reference)	
	*70–79*	2.44	(-0.59, 5.47)		0.59	(-1.92, 3.10)	
	*≥ 80*	3.39	(-1.38, 8.16)		3.39	(-0.64, 7.42)	
Health condition				< 0.001			< 0.001
	*Musculoskeletal diseases*	10.30	(7.83, 12.76)		2.47	(0.03, 4.92)	
	*Circulatory diseases*	0.00	(Reference)		0.00	(Reference)	
	*Neurological diseases*	11.28	(7.57, 15.00)		5.62	(2.44, 8.80)	
	*Neoplasms*	9.22	(4.73, 13.71)		4.37	(0.60, 8.14)	
	*Others*^*a*^^*)*^	3.84	(0.95, 6.72)		-0.86	(-3.36, 1.64)	
Multi-morbidity (ref: no)		5.50	(3.55, 7.44)	< 0.001	2.35	(0.72, 3.99)	0.005
Admission (ref: Initial)		-3,65	(-5.77, -1.52)	< 0.001	2.84	(1.02, 4.66)	0,002
Unmarried (ref: married)		2.47	(0.58, 4.36)	0.011	-0.39	(-1.97, 1.19)	0.628
Education				0.003			0.004
	*Primary school*	4.67	(1.94, 7.40)		3.66	(1.47, 5.84)	
	*Secondary school*	1.52	(-0.68, 3.71)		1.27	(-0.50, 3.04)	
	*College/university*	0.00	(Reference)		0.00	(Reference)	
Current smoking (ref: no smoking)		4.03	(1.64, 6.43)	0.001	-1.37	(-3.40, 0.67)	0.186
Rural municipality (ref: urban)		-1.03	(-2.92, 0.86)	0.283	-0.70	(-2.23, 0.83)	0.369
Physical fitness				< 0.001			< 0.001
	*Poor*	14.03	(11.47, 16.58)		5.60	(3.29, 7.90)	
	*Moderate*	6.54	(3.95, 9.14)		2.71	(0.49, 4.94)	
	*Good*	0.00	(Reference)		0.00	(Reference)	
EQ-5D (pain/discomfort)^b^^)^		5.89	(5.02, 15.52)	< 0.001	3.18	(2.28, 4.09)	< 0.001
HADS-D score^c^^)^		2.17	(1.97, 2.36)	< 0.001	1.65	(1.39, 1.91)	< 0.001
HADS-A score^d^^)^		-0.42	(-0.47, -0.38)	< 0.001	0.13	(-0.13, 0.38)	0.330

*Abbreviations*: WHODAS: World Health Organization Disability Assessment Schedule; b: unstandardized estimated regression coefficient; CI: confidence interval; ref: reference; EQ-5D: EuroQol EQ-5D; HADS-D: Hospital Anxiety and Depression scale, depression subscale; HADS-A: Hospital Anxiety and Depression scale, anxiety subscale.

^a)^ Diseases included the following: endocrine, nutritional, and metabolic diseases (n = 37), respiratory diseases (n = 36), injuries and external causes (n = 26), factors influencing health status and contact with health services (n = 23), mental and behavioural disorders (n = 13), symptoms, signs, and abnormal clinical and laboratory findings, not elsewhere classified (n = 9); codes for special purposes (n = 7); diseases of the digestive system (n = 6); diseases of the blood and blood-forming organs, and certain disorders involving the immune mechanism (n = 5); diseases of the ear and the mastoid process (n = 3); diseases of the genitourinary system (n = 3); congenital malformations, deformations, and chromosomal abnormalities (n = 3); and certain infectious and parasitic diseases (n = 2).

^b)^ From no pain/discomfort to extreme pain/discomfort, five categories.

^c)^ 0 = lowest score of depressive symptoms, 21 = highest score of depressive symptoms.

^d)^ All scores: 0 = lowest score of anxiety symptoms, 21 = highest score of anxiety symptoms.

### Predictors for domain scores

Table 3 shows the results from multivariate regression analyses for predicting WHODAS 2.0 domain scores. Most health-related variables had an effect on domain scores, with better physical fitness and psychological health resulting in less disability. The exception was for Self-care where patients who reported a moderate physical fitness scored better than patients who reported a good physical fitness. Neurological diseases significantly increased the score on disability for most domains, except for Cognition and Getting along in which neoplasms significantly increased the score. The effect of the HADS-A score varied, with significantly increased scores in Cognition, Getting along, and Participation (p < 0.05), and significantly decreased scores in Mobility, and Life activities with an increase in the HADS-A score (p < 0.001); unstandardized estimated regression coefficients ranged from −1.07 to 0.63.

**Table 3:** Results of a fully adjusted multivariate linear regression analysis for predicting WHODAS 2.0 domain scores.

Predictor variable		Cognition		Mobility		Self-care		Getting along		Life activities		Participation	
*Categories*		b	(SE)	b	(SE)	b	(SE)	b	(SE)	b	(SE)	b	(SE)
Intercept		-4.03	(2.64)	-8.41	(3.66)	-4.63	(2.74)	5.60	(3.05)	1.94	(3.99)	6.89	(2.69)
Female (ref: male)		1.27	(1.23)	1.58	(1.71)	-1.04	(1.28)	-1.98	(1.42)	9.65	(1.86)**	1.47	(1.26)
Age, years			*		**				*				*
	*18–29*	7.98	(3.44)	-2.60	(4.76)	1.65	(3.57)	3.59	(3.97)	-1.63	(5.20)	1.88	(3.50)
	*30–39*	3.29	(2.25)	-9.77	(3.11)	-1.02	(2.34)	-1.36	(2.60)	3.16	(3.40)	3.07	(2.29)
	*40–49*	5.66	(1.70)	-4.56	(2.36)	-1.23	(1.77)	5.73	(1.97)	1.50	(2.58)	3.69	(1.73)
	*50–59*	1.28	(1.53)	-5.73	(2.12)	-2.72	(1.59)	-0.28	(1.77)	-0.03	(2.32)	0.36	(1.56)
	*60–69*	0.00	(Ref)	0.00	(Ref)	0.00	(Ref)	0.00	(Ref)	0.00	(Ref)	0.00	(Ref)
	*70–79*	-1.40	(1.86)	2.58	(2.27)	0.74	(1.94)	-1.32	(2.15)	-0.90	(2.82)	-3.20	(1.90)
	*≥ 80*	-2.15	(3.22)	17.66	(4.46)	3.37	(3.35)	-1.97	(3.72)	1.37	(4.87)	-0.50	(3.28)
Health condition			*		**		*				**		
	*Musculoskeletal diseases*	-0.90	(1.78)	9.51	(2.46)	0.60	(1.84)	-0.31	(2.05)	8.36	(2.68)	1.24	(1.81)
	*Circulatory diseases*	0.00	(Ref)	0.00	(Ref)	0.00	(Ref)	0.00	(Ref)	0.00	(Ref)	0.00	(Ref)
	*Neurological diseases*	0.74	(2.32)	15.95	(3.21)	4.37	(2.41)	2.71	(2.68)	13.17	(3.51)	4.49	(2.36)
	*Neoplasms*	7.28	(2.67)	3.09	(3.69)	-1.84	(2.77)	6.78	(3.08)	8.73	(4.04)	3.45	(2.72)
	*Other*^*a*^^*)*^	-1.96	(1.81)	3.01	(2.50)	-2.55	(1.88)	-1.26	(2.09)	-1.65	(2.73)	-0.97	(1.84)
Multi-morbidity (ref: no)		2.70	(1.17)*	1.37	(1.62)	3.31	(1.22)*	1.88	(1.36)	4.74	(1.77)*	2.49	(1.19)*
Admission (ref: Initial)		1.14	(1.32)	5.34	(1.82)*	4.67	(1.37)**	-0.21	(1.52)	5.13	(1.99)*	1.96	(1.34)
Unmarried (ref: married)		-0.96	(1.14)	0.67	(1.59)	0.42	(1.19)	-1.13	(1.32)	0.77	(1.73)	-1.04	(1.16)
Education			*		**								
	*Primary school*	5.12	(1.57)	9.13	(2.17)	2.57	(1.63)	-1.43	(1.82)	1.49	(2.38)	3.62	(1.60)
	*Secondary school*	2.36	(1.27)	2.85	(1.76)	1.39	(1.32)	-0.47	(1.47)	2.15	(1.93)	2.03	(1.30)
	*College/university*	0.00	(Ref)	0.00	(Ref)	0.00	(Ref)	0.00	(Ref)	0.00	(Ref)	0.00	(Ref)
Current smoking (ref: no smoking)		-2.07	(1.46)	-1.28	(2.02)	-1.21	(1.52)	-0.44	(1.69)	-2.62	(2.21)	-2.01	(1.48)
Rural municipality (ref: urban)		0.03	(1.10)	-1.79	(1.53)	-1.03	(1.14)	0.01	(1.27)	-3.32	(1.67)*	-0.85	(1.12)
Physical fitness					**		**				**		**
	*Poor*	0.92	(1.65)	12.62	(2.29)	2.37	(1.72)	0.93	(1.91)	10.20	(2.50)	5.25	(1.68)
	*Moderate*	0.47	(1.59)	4.54	(2.21)	-0.62	(1.66)	-0.29	(1.84)	3.68	(2.41)	2.79	(1.62)
	*Good*	0.00	(Ref)	0.00	(Ref)	0.00	(Ref)	0.00	(Ref)	0.00	(Ref)	0.00	(Ref)
EQ-5D (pain/discomfort)^b^^)^		0.42	(0.65)	7.32	(0.90)**	3.00	(0.68)**	1.24	(0.75)	4.53	(0.99)**	3.86	(0.66)**
HADS-D score^c^^)^		1.91	(0.19)**	1.26	(0.26)**	0.81	(0.19)**	2.26	(0.22)**	2.53	(0.28)**	2.13	(0.19)**
HADS-A score^d^^)^		0.65	(0.18)**	-0.76	(0.25)*	-0.12	(0.19)	0.57	(0.21)*	-0.96	(0.28)**	0.47	(0.19)*

*Abbreviations*: WHODAS: World Health Organization Disability Assessment Schedule; b: unstandardized estimated regression coefficient; SE: standard error; ref: reference; EQ-5D: EuroQol EQ-5D; HADS-D: Hospital Anxiety and Depression scale, depression subscale; HADS-A: Hospital Anxiety and Depression scale, anxiety subscale.

^a)^ Diseases included the following: endocrine, nutritional, and metabolic diseases (n = 37), respiratory diseases (n = 36), injuries and external causes (n = 26), factors influencing health status and contact with health services (n = 23), mental and behavioural disorders (n = 13), symptoms, signs, and abnormal clinical and laboratory findings, not elsewhere classified (n = 9); codes for special purposes (n = 7); diseases of the digestive system (n = 6); diseases of the blood and blood-forming organs, and certain disorders involving the immune mechanism (n = 5); diseases of the ear and the mastoid process (n = 3); diseases of the genitourinary system (n = 3); congenital malformations, deformations, and chromosomal abnormalities (n = 3); and certain infectious and parasitic diseases (n = 2).

^b)^ From no pain/discomfort to extreme pain/discomfort, five categories.

^c)^ 0 = lowest score of depressive symptoms, 21 = highest score of depressive symptoms.

^d)^ All scores: 0 = lowest score of anxiety symptoms, 21 = highest score of anxiety symptoms.

*p ≤ 0.05

**p ≤ 0.001.

## Discussion

Rehabilitation patients have increased disability as a common characteristic. The mean overall WHODAS 2.0 disability score among patients who were accepted for admission to a rehabilitation center was 31.6 for women and 27.3 for men. In the normal non-institutionalized population in adults older than 18 years and in those living in private households, a score of 30 corresponds to the score for the 88th population percentile [26].

In the present study, disability scores for each domain varied considerably, and an important finding was that the highest disability scores among domains were found for Life activities and Participation. Although the WHODAS 2.0 may tend to favor a medical construct of disability [31], the items in Participation include explicitly contextual factors, which are seldom present in disability assessment instruments.

In our study, neurological disease, multi-morbidity, ongoing management, low educational attainment, impaired physical fitness, pain, and depressive symptoms significantly increased the overall disability score. Predictors of domain-specific disabilities varied with these variables, in addition to sex, age, urbanicity, and symptoms of anxiety. Marital status and smoking were the only variables that were not associated with any domain.

### Strengths and limitations

This study has several important strengths. This is the first large cohort study in Norway among patients accepted for admission to a rehabilitation center, including nearly one thousand participants, representing the most common diagnoses that are found in the rehabilitation services. All patients from the western part of Norway, which includes 21.0% of the country’s inhabitants [32], who were accepted for admission to a rehabilitation center within the first half of 2015, were invited to participate. The study was based on a large number of validated survey instruments and information from referral letters that was merged with data from public registers. There was a low number of missing values.

The main limitation is a relatively low response rate, probably due to the high number of items in the survey, leading to some selection bias. Although the response rate weakens the representativeness of the study sample and the external validity, the investigation of predictors relating to disability should be valid. The cross-sectional study design only presents associations and cannot explain the direction of causality. The external validity of findings is affected by the setting and dependent on Norwegian regulations determining the practice of rehabilitation in secondary care.

### Possible explanations for the present findings and comparison with previous studies

The overall disability score as assessed by the WHODAS 2.0 of approximately 30 out of the maximum of 100, may be regarded as a relatively high functional level, considering 45 as the limit for substantial disability [33]. However, the present study showed higher overall disability scores than those previously found in similar studies [34, 35]. However, comparing absolute disability scores may be challenging because of contextual factors, including different health systems and time trends. The overall disability scores disguise larger domain-specific variations and the clinical utility of a sum score is questionable because various disability domains are included in this score. Consequently, the predictor variables are discussed and explained primarily in terms of domains.

In practice, criteria for admission to rehabilitation centers differ according to age. The requirements for older patients are stricter and a clear potential for improvement must be present, excluding the most disabled older people, both physically and cognitively. Consequently, increased disability with increasing age, which has been previously reported [3–5], was not found in the present study for the overall disability score. However, higher age was associated with higher disability for Mobility and lower disability for Cognition.

In our study, the score of Self-care, which assesses items addressing hygiene, dressing, eating, and staying alone, was especially low because most patients have to be able to care for themselves in the rehabilitation centers. In terms of some domain-specific disability scores, this contributes to a relatively homogenous study sample.

Despite the extended safety net of social welfare services in Norway aiming to enable participation in society, the score of Participation was high. The domain of Participation addresses contextual factors, including facilitation, others’ attitudes and actions, and family and economic consequences of health conditions. These factors apply to various aspects of the social structure and are traditionally not targeted by health services. However, a more comprehensive understanding of this domain and the contextual factors influencing it, may contribute to improvement of interventions. This applies to all health conditions because scores in this domain were not associated with health conditions in the adjusted model in our study. The association between Participation and physical fitness may be related to physical barriers in society. A higher educational level has a universally positive effect on all forms of civic and social engagement [36], which may explain the lower scores in Participation.

In previous studies, women generally scored higher on disability [6–11], which was not found in the present study. The only exception was higher scores in Life activities among women after adjustments, revealing problems concerning work and household. This probably reflects the traditional gender roles with less male responsibilities for the household [11].

In our study, the scores of disability varied between health conditions, which is in concordance with previous studies [11, 22, 37]. The scores for neurological diseases were especially high for domains mainly including physical components, Mobility and Life activities, which is consistent with a previous study [22]. An interesting finding in our study was the relation between neoplasms and high scores in the cognitive domains of Cognition and Getting along. Cognitive difficulties have been reported for patients after cancer treatment [38, 39] and should be taken into consideration when planning rehabilitation interventions for this group.

Multi-morbidity has been found to increase the level of disability [7, 13, 40], which was also found in the present study. However, there was no association between multi-morbidity and Mobility. This is in contrast to a previous study that assessed domain-specific associations [11], and in studies that only used instruments capturing mobility [13, 40]. One explanation for this discrepancy between our study and other studies may be the exclusion of the most disabled patients with high multi-morbidity in Norwegian rehabilitation centers. While our study investigated multi-morbidity as a dichotomized variable, other studies used several categories [11] or used multi-morbidity as a continuous variable [7], where a gradual increase in disability with the number of chronic conditions was reported.

In rehabilitation programs, physical activities are often included to increase the health and function of patients [41]. A previous study on adults with arthritis [14] showed that better perceived physical health was associated with lower disability levels. With regard to overall disability, this finding is in accordance with our study. However, only scores for domains including physical components were associated with this variable. For domains with mainly cognitive components, Cognition and Getting along, no effect of perceived physical fitness was observed.

Higher pain increases the disability scores in people with arthritis [14], which is in agreement with findings in the present study. Although a reduction in pain is not usually considered as the primary goal of rehabilitation, a reduction in pain may be a secondary gain, intervening with cognitive and physical components. In this study, pain did not affect disability scores in the cognitive domains of Cognition and Getting along.

In our study, depressive symptoms significantly increased disability in all domains, which is consistent with studies that included disease-specific groups and older people [15, 40].

In the current study, no significant association between symptoms of anxiety and the overall disability score was found. However, more symptoms of anxiety increased disability in Cognition, Getting along, and Participation, but resulted in lower scores in Mobility, Self-care, and Life activities. The role of anxiety has been less investigated compared with depressive symptoms, but is associated with disability in older populations only in women [7]. Whether this finding reflects a significant association or merely a statistical artefact should be further investigated in larger population studies. A previous prospective cohort study showed that disability per se predicts future disability for older people [42]. This variable should be investigated further in a prospective study and a regression model for analysis of future disability in the present cohort is currently underway.

The WHODAS 2.0 does not assess all aspects of disability, and the results may not necessarily correlate with the specific disability for which for the patient is referred. However, we assume that the most important aspects of disability are included in the WHODAS 2.0 and are of significance for each patient.

## Conclusion

The present study shows a relatively low overall disability level, which is probably explained by the fact that most patients must be able to care for themselves in the rehabilitation centers in the secondary care in Norway. Patients struggle most in Life activities and Participation, and this should be taken into account when future treatment strategies for rehabilitation services are developed. However, targeting these domains can also be done in primary care, probably even better, because of the competence related to the local community. Further research to identify determinants for disability should especially focus on participation restrictions to improve rehabilitation as a comprehensive process. Knowledge of and targeting determinants of disability may not only reduce disability levels, but could also improve other clinical outcomes, such as quality of life.

Referring patients with lower level of disability to primary care could allocate more resources for rehabilitation to patients with higher level of disability at the rehabilitation centers. However, the potential of improvement following rehabilitation as a criterion for admission must not be waived.
